# Potential Value of Serum Uric Acid in the Identification of Postoperative Delirium in Geriatric Patients Undergoing Knee Replacement

**DOI:** 10.3389/fnagi.2022.909738

**Published:** 2022-07-14

**Authors:** Fei Wang, Xinhui Tang, Jiahan Wang, Siyv Liu, Xiaoyue Wu, Rui Dong, Xu Lin, Bin Wang, Yanlin Bi

**Affiliations:** ^1^Department of Anesthesiology, Qingdao Municipal Hospital Affiliated to Qingdao University, Qingdao, China; ^2^Department of Anesthesiology, Dalian Medical University, Dalian, China; ^3^Department of Anesthesiology, Weifang Medical College, Weifang, China; ^4^Department of Anesthesiology, Drum Tower Hospital Affiliated to Nanjing University Medical School, Nanjing, China

**Keywords:** Alzheimer-related biomarkers, risk factor, prediction, postoperative delirium, serum uric acid

## Abstract

**Objectives:**

The relationship between preoperative serum uric acid (SUA) and cerebrospinal fluid (CSF) Alzheimer-related biomarkers were investigated to determine whether high SUA is a potential risk factor for postoperative delirium (POD) and to evaluate its predictive efficacy.

**Methods:**

The participants were selected from the Perioperative Neurocognitive Disorder Risk Factor and Prognosis (PNDRFAP) study and the Perioperative Neurocognitive Disorder and Biomarker Lifestyle (PNDABLE) study. The logistic regression equation was used to analyze the risk factors and protective factors of POD. The interaction term (SUA × Sex) was introduced into the linear model to explore the potential modification effects of sex on the identified correlations. We analyzed the mediating effects of Alzheimer-related biomarkers. Finally, we constructed the receiver operating characteristic (ROC) curve and the nomogram model to evaluate the efficacy of SUA and Alzheimer-related biomarkers in predicting POD.

**Results:**

Patients with POD had elevated SUA level (PNDRFAP: *p* = 0.002, PNDABLE: *p* < 0.001). Preoperative SUA level was positively correlated with CSF phosphorylated tau (P-tau) (*p* = 0.027) and β-amyloid42 (Aβ_42_)/P-tau (*p* = 0.023). Interaction analysis did not find any modification effect of sex. The relationship between SUA and POD was partially mediated by CSF P-tau (15.3%). ROC curve showed that the model combining SUA and Alzheimer-related biomarkers had better performance in predicting POD [area under the curve (AUC) = 0.880; *p* < 0.001], and the predictive model is accurate.

**Conclusions:**

High SUA may enhance CSF P-tau level, thus increasing the risk of POD, and the model combining SUA and Alzheimer-related biomarkers can accurately predict the occurrence of POD.

## Introduction

Postoperative delirium (POD), an acute and temporary central nervous system (CNS) dysfunction that usually occurs within 3–5 days after surgery, is going to exert adverse effects on the prognosis of patients (Oh and Park, 2019). Currently, the exact pathogenesis of onset of POD remains controversial and the main treatment for POD is drug therapy, such as haloperidol and dexmedetomidine (Popp and Arlt, 2012; Hayhurst et al., 2016), but due to insufficient evidence of efficacy or significant side effects, further studies are needed to evaluate the impact of drug therapy on patients' long-term prognosis. Early detection and treatment may be a new way to reduce the incidence and consequences of delirium (Hayhurst et al., 2016). Hence, clarifying risk factors for POD onset is a very important theme in clinical practice.

Accordingly, a variety of clinical factors have been proposed for assessing the risk of POD occurrence. The roles of the Alzheimer-related biomarkers in cerebrospinal fluid (CSF), such as β-amyloid40 (Aβ_40_), β-amyloid42 (Aβ_42_), total tau (T-tau), and phosphorylated tau (P-tau), have been largely addressed in inducing neurological abnormalities (DaRocha-Souto et al., 2011; Körtvélyessy et al., 2015; Wang and Mandelkow, 2016) and the core proteins of POD (Sun et al., 2016; Huang et al., 2018). Aβ interferes with synaptic function by binding to different components of the neuronal or non-neuronal plasma membrane, which is the basis of clinical manifestations of cognitive decline (Mucke and Selkoe, 2012). After tau protein is phosphorylated, it loses the function of stabilizing the microtubule cytoskeleton, thus causing neurodegenerative diseases (Nizynski et al., 2017).

The imbalance of metabolism in CNS is generally associated with several pathogeneses, and the identification of metabolites might be reflective of CNS dysfunction. Uric acid is the final product of purine metabolism. An emerging body of evidence suggests that serum uric acid (SUA) level is one of the factors affecting cognitive function (Beydoun et al., 2016; Alam et al., 2020). SUA was reported to be neuroprotective *via* reducing the oxidation of natural low-density lipoprotein (LDL) and enhancing the oxidation effect of oxidized LDL by the presence of transition metals (Bagnati et al., 1999; So and Thorens, 2010). In addition, SUA is also associated with several cardiovascular and cerebrovascular diseases, which may be negatively correlated to cognitive function (Borghi et al., 2015). However, whether SUAs were correlated to Alzheimer-related biomarkers and their roles on POD were not fully discussed.

In this study, we aimed to explore SUA levels in patients with POD and their relationship with CSF Alzheimer-related biomarkers, so as to determine whether high SUA is a potential risk factor for POD. We designed the following steps: (1) The difference in uric acid levels between POD and non-POD patients was investigated to evaluate whether high SUA is a risk factor for POD; (2) To investigate the association between SUA and POD.

## Methods

### Study Enrollment

Data for this study were obtained from voluntary participants in the Perioperative Neurocognitive Disorder Risk Factor and Prognosis (PNDRFAP) study between January 2020 and May 2021 (Deng et al., 2022), and the Perioperative Neurocognitive Disorder and Biomarker Lifestyle (PNDABLE) study between November 2020 and December 2021 (Lin et al., 2021; Wang et al., 2021).

This study was approved by the Ethics Committee of Qingdao Municipal Hospital (Clinical registration number of PNDRFAP: ChiCTR2000033639, PNDABLE: ChiCTR2000033439). CSF samples were collected from all participants after written informed consent was obtained from the patients or their legal representatives.

In the PNDRAFP study, the inclusion criteria included: (1) Patients between 50 and 90 years; (2) American Society of Anesthesiologists (ASA) physical status classification system I-II; (3) Patients who had intact preoperative cognitive function without communication disorders; and (4) Patients who had sufficient education to complete the preoperative neuropsychological tests. Exclusion criteria included: (1) Mini-Mental State Examination (MMSE) score of <24; and (2) Patients with serious psychological disorders or hearing impairment.

In the PNDABLE study, the inclusion criteria included: (1) Patients between 50 and 90 years; (2) ASA I-II; (3) Undergoing total knee arthroplasty; and (4) Combined spinal-epidural anesthesia was intended. Exclusion criteria included: (1) Preoperative MMSE score of <24; (2) Drug or psychotropic substance abuse, as well as long-term use of steroid drugs and hormone drugs; (3) Recent major surgery; (4) Severe visual and hearing impairments; (5) Abnormal coagulation function before surgery; (6) CNS infection, head trauma, multiple sclerosis, neurodegenerative diseases other than Alzheimer's disease (AD), or other major neurological disorders; (7) Major psychological disorders; (8) Severe systemic diseases that may affect CSF or blood levels of Alzheimer-related biomarkers including Aβ and tau protein; and (9) Family history of genetic diseases.

**Figure 1:**
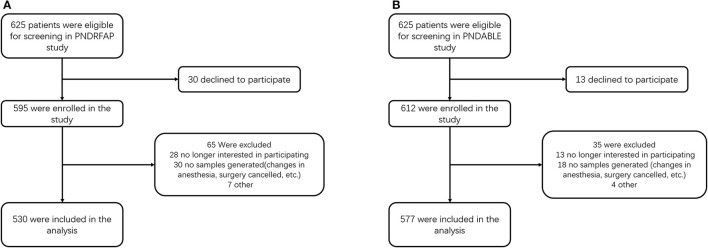
Diagram of study design. **(A)** The Perioperative Neurocognitive Disorder Risk Factor and Prognosis (PNDRFAP) study. **(B)** The Perioperative Neurocognitive Disorder and Biomarker Lifestyle (PNDABLE) study.

The preliminary test found that 5 covariates were expected to enter the logistic regression, the POD incidence was 10%, and the loss of follow-up rate was assumed to be 20%, so the required sample size was calculated to be 625 cases [5 × 10 ÷ 10% ÷ (1–20%)]. After screening, 530 people were included in PNDRFAP and 577 were included in PNDABLE (Figure 1).

### Anesthesia and Surgery

The participants did not receive preoperative medications, and they were instructed not to drink for 6 h and not to eat for 8 h before surgery. After entering the operating room, we routinely monitored ECG, Pulse Oxygen Saturation (SpO2), and Noninvasive Blood Pressure (NBP), opened vein access and extracted 3 ml of whole venous blood.

Patients in the PNDRFAP study received general anesthesia. Anesthesia induction used Sufentanil 0.2–0.5 μg/kg, Ceftriaxonium 0.15–0.2 mg/kg, and Etomidate 0.15–0.3 mg/kg. Continuous pumping of Remifentanil 0.25–2 μg/kg·min maintained intraoperative analgesia. Cis-atracurium was injected every 40 min after induction and stopped 1 h before the end of surgery. Sevoflurane is inhaled 0.5–3% depending on the depth of anesthesia.

Patients in the PNDABLE study underwent combined spinal-epidural anesthesia. The anesthesia position was lateral decubitus, with the space between the spinous processes of lumbar 3–4 (L_3_-L_4_) as the puncture site. After the successful puncture, 2 ml of CSF was extracted from the subarachnoid space, followed by an injection of 2–2.5 ml Ropivacaine (0.66%) for about 30 s. CSF samples were centrifuged for 10 min at 2,000×*g* and the CSF supernatant was stored in an enzyme-free EP (Eppendorf) tube (AXYGEN; PCR-02-C) at −80°C for subsequent studies. After anesthesia, the sensory level was controlled below the thoracic 8 (T_8_) level. During the surgery, oxygen was inhaled via mask at 5 l/min and vasoactive drugs were administered moderately to maintain the vital signs of the patients at a stable level.

The patient was sent to the anesthesia resuscitation room after the operation, observed for 30 min, and sent back to the ward if there was no abnormality. Intravenous patient-controlled analgesia (Butorphanol tartrate injection 10 mg + Toranisetron hydrochloride injection 5 mg + 0.9% sodium chloride solution 89 ml maintained pain digital score < 3 points) was used in postoperative pain management.

### Data Collection

A total of 3 ml of fasting venous blood was taken before surgery and the SUA level was determined by enzyme coupling assay. Using clinical diagnostic criteria for hyperuricemia (Lin et al., 2000), the population was divided into hyperuricemia group (HS) (male ≥416.5 μmol/l, female ≥357 μmol/l) and normal uric acid group (NS) (male < 416.5 μmol/l, female < 357μmol/l).

### Measurements of CSF Sampling

Cerebrospinal fluid (CSF) Alzheimer-related biomarkers were measured by enzyme-linked immunosorbent assay (ELISA) using the microplate reader (Thermo Scientific Multiskan MK3). All SUA samples in this study were analyzed by experienced technicians using the same methods. The measurements were performed in triplicate and averaged for statistical analysis.

### Neuropsychological Tests

The preoperative cognitive status was assessed by neurologists using the MMSE. Those patients with MMSE scores <24 points were excluded. The delirium assessment was performed two times a day at 9:00–10:00 am and 2:00–3:00 pm on 1–7 days (or before discharge) by an anesthesiologist postoperatively. POD was defined by the Confusion Assessment Method (CAM) (Inouye et al., 1990). The diagnostic criteria for POD consist of four clinical criteria: (1) Acute onset and fluctuating course; (2) Inattention; (3) Disorganized thinking; (4) Altered level of consciousness. For delirium to be defined, both (1) and (2) have to be met concurrently, plus either (3) and/or (4). POD severity was measured using the Memorial Delirium Assessment Scale (MDAS) (De J, 2015). According to whether POD occurred or not, the participants were divided into POD group (POD) and no postoperative delirium group (NPOD).

### Statistical Analysis

The data were analyzed using Stata MP16.0 (Solvusoft Corporation, Inc., Chicago, Illinois, USA), GraphPad Prism version 7.0 (GraphPad Software, Inc., LaJolla, CA, USA), and R software version 4.4.1 (R Foundation for Statistical Computing, Vienna, Austria). For demographics, the Kolmogorov–Smirnov test was used to determine whether the measured data were normally distributed. The *t*-test and the Mann–Whitney U test were used for continuous variables, and the chi-square test/Fisher's exact test was used for categorical variables.

The logistic regression equation was used to analyze the risk factors and protective factors of POD. In order to eliminate the influence of confounding factors, we conducted multiple sensitivity analyses in this study and three correction models were constructed: (1) Age, sex, years of education, and MMSE score were included; (2) In addition to the above factors, the history of hypertension, diabetes, smoking, and drinking were also included for correction; and (3) On the basis of (2), age ≥65 is further restricted. Sex-based clinical diagnostic criteria for hyperuricemia were used to further validate the findings.

The interaction effect of sex was analyzed to exclude its interference with the association of SUA and the Alzheimer-related biomarkers in CSF. The linear model was constructed to test the interaction terms between SUA and sex.

Moreover, linear regression models covering three equations were performed to examine whether the association between SUA and POD was mediated by Alzheimer-related biomarkers in CSF (Liu and Ulrich, 2016). Mediation effects were established if the following criteria were simultaneously reached: (1) Changes in SUA were significantly affect the Alzheimer-related biomarkers in CSF; (2) Changes in Alzheimer-related biomarkers in CSF were account for variations in the POD; (3) Changes in SUA were significantly or not significantly related to POD; and (4) The association between SUA and POD was attenuated when the Alzheimer-related biomarkers were added in the regression model. Furthermore, the attenuation or indirect effect was estimateVd, with the significance determined using 10,000 bootstrapped iterations, where each path of the model was controlled for age, sex, years of education, and MMSE.

The predictive value of SUA and the Alzheimer-related biomarkers was described with a receiver-operating characteristics (ROC) curve and the area under the curve (AUC) reported the discriminatory ability. Calibration was used to verify the predicted model. Furthermore, we established a nomogram incorporating these independent variables.

*p* < 0.05 was considered significant.

## Results

### Characteristics of Participants

In the PNDRFAP cohort, a total of 530 participants were included according to study criteria (Figure 1A). No significant differences were observed in age, sex, years of education, MMSE scores, history of hypertension, and diabetes between the HS and NS. Those who had a history of smoking and drinking presented higher SUA levels. The results showed a higher incidence and severity of POD in the HS (Table 1).

**Table 1:** Characteristics of participants in PNDRFAP and PNDABLE.

	**PNDRFAP**			**PNDABLE**		
**Characteristic**	**NS^a^ (*n =* 405)**	**HS (*n =* 125)**	***P***	**NS (*n =* 461)**	**HS (*n =* 116)**	***P***
Age [year, M(Q)]	67 (13)	65 (19)	0.355	62 (14)	68 (17)	0.000^*^
Male [*n* (%)]	232 (57.28)	80 (64)	0.182	292 (63.34)	53 (45.69)	0.001^*^
Education [year, M(Q)]	9 (2)	9 (5)	0.497	9 (3)	9 (4)	0.581
MMSE [scores, M(Q)]	26 (1.50)	26 (3)	0.751	28 (3)	28 (2)	0.098
Smoking history [*n* (%)]	141 (34.81)	59 (47.20)	0.013^*^	141 (30.59)	25 (21.55)	0.055
Drinking history [*n* (%)]	122 (30.12)	54 (43.20)	0.007^*^	167 (36.23)	31 (26.72)	0.054
Hypertension [*n* (%)]	213 (52.59)	69 (55.20)	0.610	158 (34.27)	61 (52.59)	0.000^*^
Diabetes [*n* (%)]	112 (27.65)	32 (25.60)	0.625	74 (16.05)	30 (25.86)	0.014^*^
SUA [μmol/l, M(Q)]	294.76 (83.31)	464.37 (96.39)	0.000^*^	291.87 (99)	426.63 (87)	0.000^*^
POD [*n* (%)]	66 (16.30)	32 (25.60)	0.019^*^	71 (15.40)	58 (50)	0.000^*^
MDAS [*n* (%)]	3 (5)	4 (6)	0.000^*^	2 (5)	8.5 (13)	0.000^*^
Aβ42 [pg/ml, M(Q)]	–	–	–	352.18 (283.07)	302.94 (217.38)	0.066
P-tau [pg/ml, M(Q)]	–	–	–	38.52 (22.46)	42.06 (37.67)	0.027^*^
T-tau [pg/ml, M(Q)]	–	–	–	208.54 (130.28)	209.79 (158.99)	0.790
Aβ_42_/P-tau [M(Q)]	–	–	–	8.71 (7.44)	7.36 (7.91)	0.023^*^
Aβ_42_/T-tau [M(Q)]	–	–	–	1.67 (1.69)	1.53(1.65)	0.082

*PNDRFAP, the Perioperative Neurocognitive Disorder Risk Factor and Prognosis study; PNDABLE, the Perioperative Neurocognitive Disorder and Biomarker Lifestyle study; NS, Normal SUA; HS, High SUA; SUA, serum uric acid; POD, postoperative delirium; MDAS, the Memorial Delirium Assessment Scale; Aβ_42_, β-amyloid42; T-tau, total tau; P-tau, phosphorylated tau*.

*^a^NS: male <416.5μmol/l, female <357μmol/l, HS: male ≥ 416.5μmol/l, female ≥ 357μmol/l*.

*Continuous variable use Student's t test or Mann-Whitney U, Categorical variable use Chi-square test*.

*The numerical variables of normal distribution are statistically described by Average ± Standard deviation [x̄ ± s]*.

*Non-normally distributed numerical variables are statistically described by Median (Interquartile spacing) [M(Q)]*.

*Categorical variables are statistically described by Sample size (Percent) [n (%)]*.

* *P < 0.05*.

**Figure 2:**
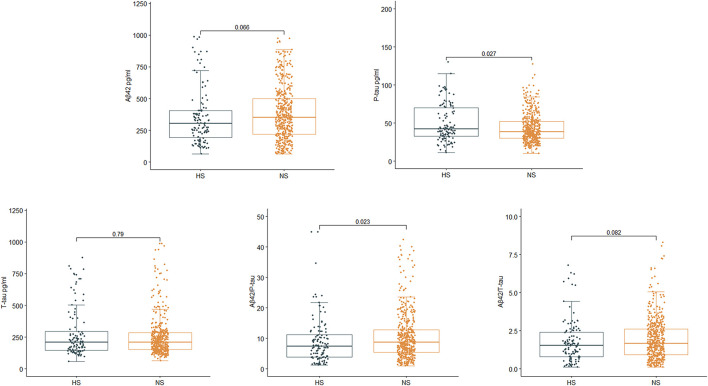
Relationship between serum uric acid (SUA) and cerebrospinal fluid (CSF) Alzheimer-related biomarkers in PNDABLE. CSF phosphorylated tau (P-tau) was positively correlated with SUA (*p* = 0.027), while CSF Aβ_42_/P-tau was negatively correlated with SUA (*p* = 0.023).

For the PNDABLE cohort, a total of 577 participants were included (Figure 1B). We detected no significant differences in years of education, MMSE scores, smoking and drinking history, CSF Aβ_42_, CSF T-tau, and CSF Aβ_42/_T-tau in participants. Compared to NS, the HS had covered more female patients with advanced age, a higher incidence of hypertension and diabetes. CSF P-tau level was higher (*p* = 0.027) and CSF Aβ_42_/P-tau level was significantly lower (*p* = 0.023) in HS (Figure 2). In addition, the incidence and severity of POD were higher in HS.

### Metabolic Changes of SUA and Alzheimer-Related Biomarkers in POD Patients

We measured the levels of uric acid and Alzheimer-related biomarkers between POD and NPOD.

**Figure 3:**
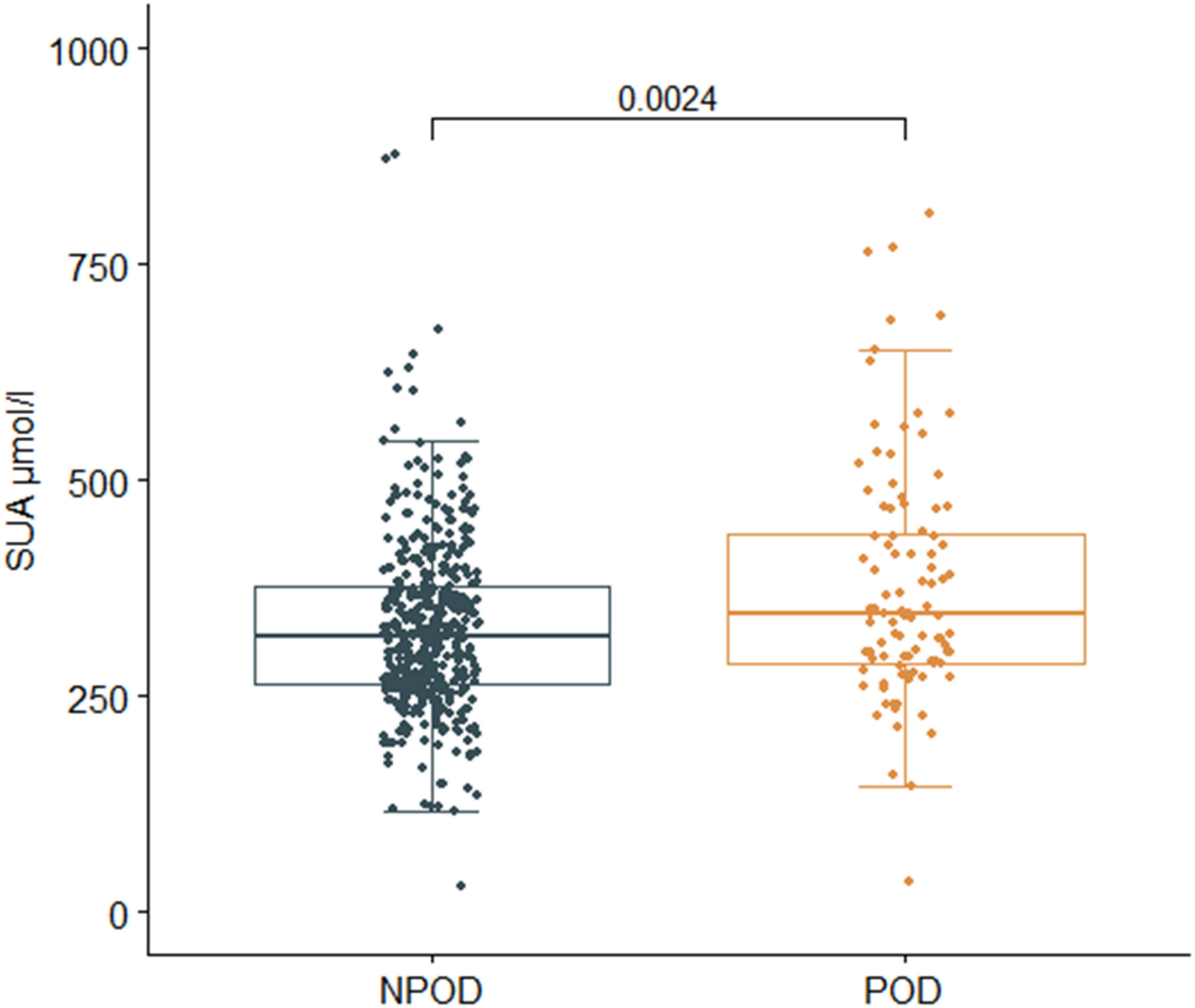
Relationship between SUA and postoperative delirium (POD) in Perioperative Neurocognitive Disorder Risk Factor and Prognosis (PNDRFAP). The incidence of POD was positively correlated with SUA (*p* = 0.002).

Results in the PNDRFAP cohort showed that patients with POD had a higher preoperative SUA level (*p* = 0.002) (Figure 3). Adjusted for age, sex, years of education, and MMSE scores (Model 2), based on a multivariate linear model, the increase of SUA was significantly associated with the occurrence of POD (odds ratio [OR]:1.004; 95% confidence interval [95% Confidence Interval (CI)]:1.001–1.007; *p* = 0.014). After adjusting for the history of hypertension, diabetes, smoking, and drinking (Model 3), the results remained robust (OR:1.004; 95% CI:1.000–1.007; *p* = 0.023). We then adjusted for age ≥65 (Model 4), and the results were similar (OR:1.004; 95% CI:1.000–1.007; *p* = 0.031) (Table 2).

**Table 2:** Logistic regression on analysis and sensitivity analysis in PNDRFAP study.

	**Model 1^a^**		**Model 2^b^**		**Model 3^c^**		**Model 4^d^**	
	**OR (95%CI)**	***P***	**OR (95%CI)**	***P***	**OR (95%CI)**	***P***	**OR (95%CI)**	***P***
SUA, μmol/L	1.004 (1.002–1.005)	0.002^*^	1.004 (1.001–1.007)	0.014^*^	1.004 (1.000–1.007)	0.023^*^	1.004 (1.000–1.007)	0.031^*^

*OR, odds ratio; 95%CI, 95% confidence interval; SUA, serum uric acid*.

a *Model 1: Unadjusted*.

b *Model 2: Adjusted for age (50–90), sex, years of education and MMSE scores*.

c *Model 3: Adjusted for age (50–90), sex, years of education, MMSE scores, smoking history, drinking history, hypertension, and diabetes*.

d *Model 4: Adjusted for age≥65, sex, years of education, MMSE scores, smoking history, drinking history, hypertension, and diabetes*.

* *P < 0.05*.

**Figure 4:**
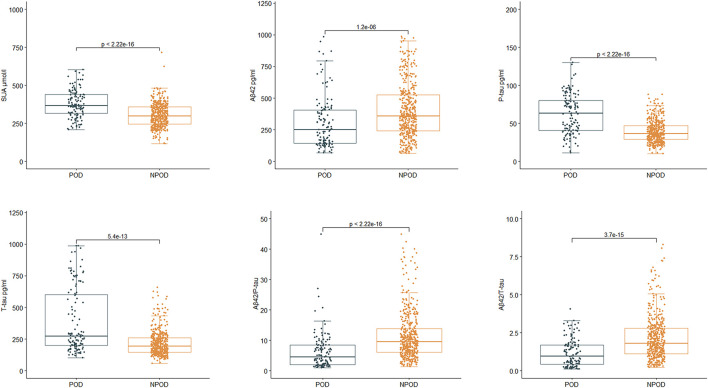
Relationship between SUA, cerebrospinal fluid (CSF) Alzheimer-related biomarkers, and POD in PNDABLE. SUA, CSF phosphorylated tau (P-tau), and CSF total tau (T-tau) were increased in POD group (*P* < 0.001), while CSF β-amyloid42 (Aβ_42_), CSF Aβ_42_/P-tau and CSF Aβ_42_/T-tau were decreased (*p* < 0.001).

In PNDABLE cohort, SUA, CSF P-tau, and CSF T-tau were increased in POD group (*p* < 0.001), while CSF Aβ_42_, CSF Aβ_42_/P-tau, and CSF Aβ_42_/T-tau were decreased (*p* < 0.001) (Figure 4). The above results remained robust in the three correction models (Model 2, Model 3, and Model 4) (Table 3).

**Table 3:** Logistic regression on analysis and sensitivity analysis in PNDABLE study.

	**Model 1^a^**		**Model 2^b^**		**Model 3^c^**		**Model 4^d^**	
	**OR (95% CI)**	***P***	**OR (95% CI)**	***P***	**OR (95% CI)**	***P***	**OR (95% CI)**	***P***
SUA, μmol/l	1.011 (1.009–1.014)	0.000^*^	1.013 (1.009–1.017)	0.000^*^	1.013 (1.009–1.017)	0.000^*^	1.015 (1.010–1.020)	0.000^*^
Aβ_42_, pg/ml	0.998 (0.997–0.999)	0.000^*^	0.999 (0.997–1.000)	0.039^*^	0.998 (0.997–1.000)	0.035^*^	0.999 (0.997–1.000)	0.049^*^
P-tau, pg/ml	1.061 (1.049–1.074)	0.000^*^	1.053 (1.036–1.070)	0.000^*^	1.054 (1.037–1.072)	0.000^*^	1.058 (1.039–1.077)	0.000^*^
T-tau, pg/ml	1.006 (1.004–1.007)	0.000^*^	1.005 (1.003–1.007)	0.000^*^	1.005 (1.003–1.007)	0.000^*^	1.005 (1.003–1.007)	0.000^*^
Aβ_42_/P-tau	0.849 (0.809–0.892)	0.000^*^	0.888 (0.846–0.933)	0.000^*^	0.886 (0.843–0.931)	0.000^*^	0.888 (0.845–0.934)	0.000^*^
Aβ_42_/T-tau	0.426 (0.334–0.544)	0.000^*^	0.557 (0.412–0.752)	0.000^*^	0.561 (0.412–0.763)	0.000^*^	0.565 (0.414–0.771)	0.000^*^

*OR, odds ratio; 95%CI, 95% confidence interval; SUA, serum uric acid; Aβ_42_, β-amyloid42; T-tau, total tau; P-tau, phosphorylated tau*.

a *Model 1: Unadjusted*.

b *Model 2: Adjusted for age (50–90), sex, years of education and MMSE scores*.

c *Model 3: Adjusted for age (50–90), sex, years of education, MMSE scores, smoking history, drinking history, hypertension, and diabetes*.

d *Model 4: Adjusted for age≥65, sex, years of education, MMSE scores, smoking history, drinking history, hypertension, and diabetes*.

* *P < 0.05*.

### Analysis of Sex Interaction Effects

The interaction analysis found no modification effect of sex, indicating that the association between SUA and the Alzheimer-related biomarkers was independent of sex (Table 4).

**Table 4:** Transactional analysis in PNDABLE.

	**SUA × Sex**	
**Alzheimer-related biomarkers**	***F***	***P***
Aβ_42_, pg/ml	2.409	0.124
P-tau, pg/ml	0.328	0.904
T-tau, pg/ml	0.187	0.972
Aβ_42_/P-tau	1.390	0.324
Aβ_42_/T-tau	0.578	0.740

*SUA, serum uric acid; Aβ_42_, β-amyloid42; T-tau, total tau; P-tau, phosphorylated tau*.

### Causal Mediation Analysis

**Figure 5:**
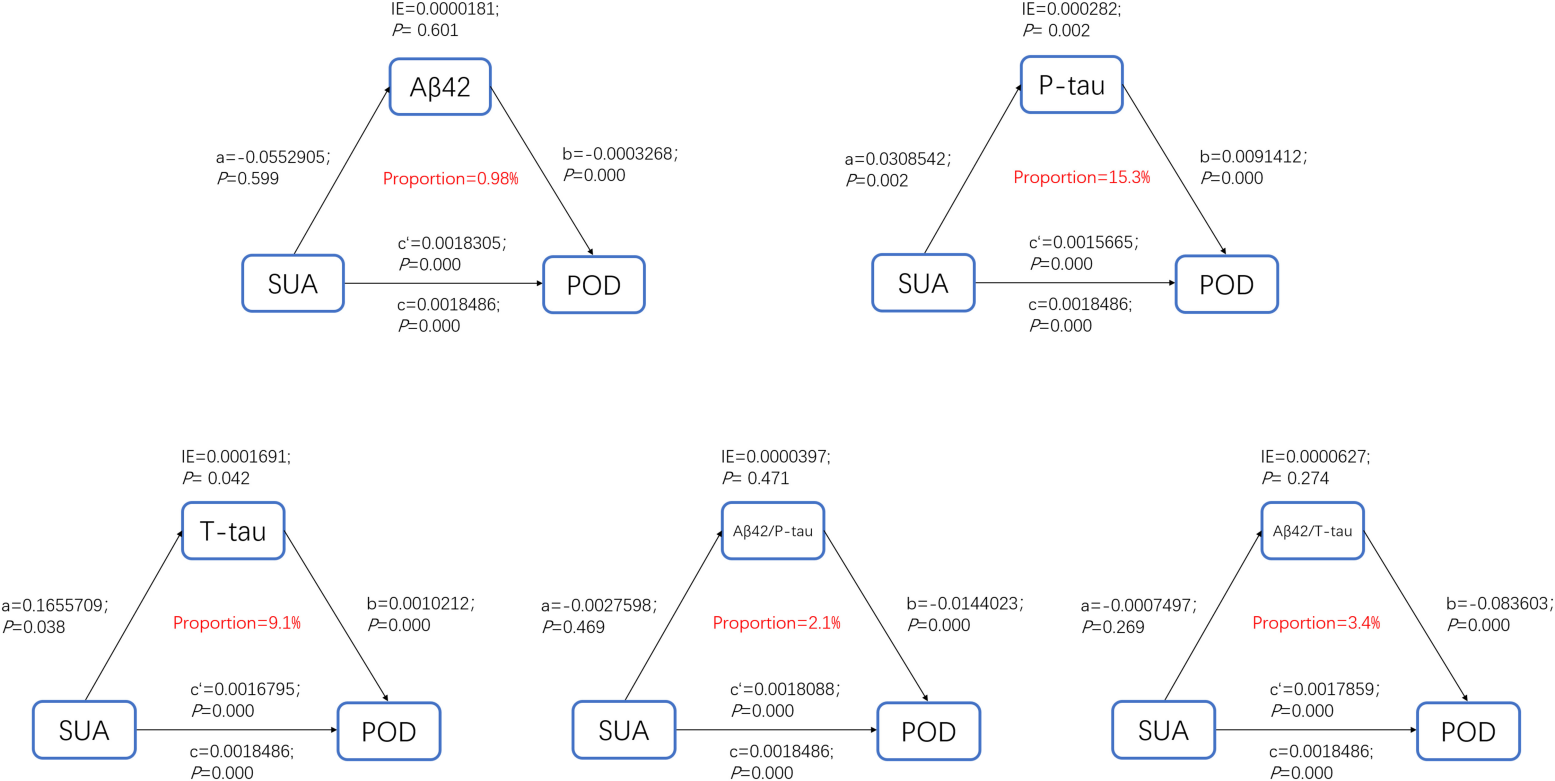
Mediation analysis. The relationship between SUA and POD was mediated by CSF P-tau (proportion = 15.3%).

The above findings suggested that high SUA was not only a significant risk factor for POD but also a potential modulator of the Alzheimer-related biomarkers. We next investigated whether SUA contributes to POD *via* modulating the Alzheimer-related biomarkers, and found that the relationship between SUA and POD was mediated by CSF P-tau (proportion = 15.3%) (Figure 5).

### Predictive Model

**Figure 6:**
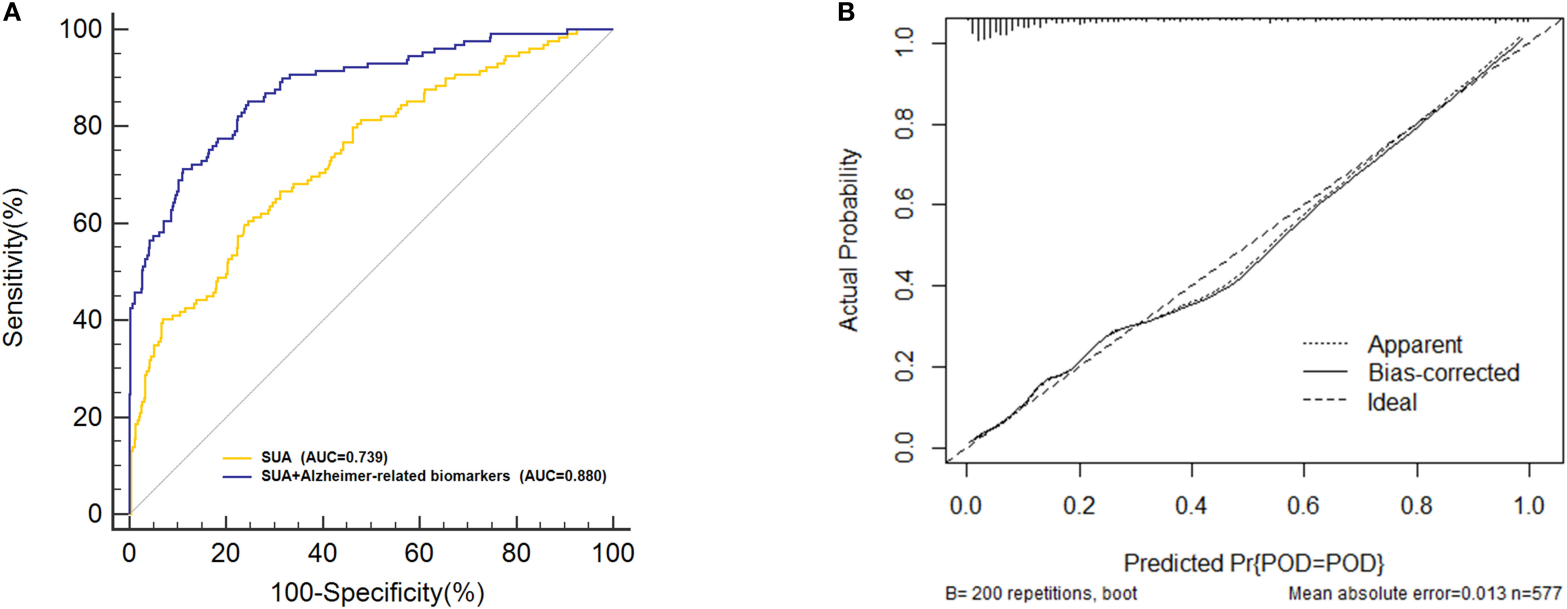
Predictive model. **(A)** The model combining serum uric acid (SUA) and the Alzheimer-related biomarkers [area under the curve (AUC) = 0.880; *p* < 0.001] exhibited a relative better discriminatory ability in postoperative delirium (POD) prediction compared to only SUA (AUC = 0.739; *p* < 0.001). **(B)** Calibration curve demonstrated the effectiveness of the predictive model.

**Figure 7:**
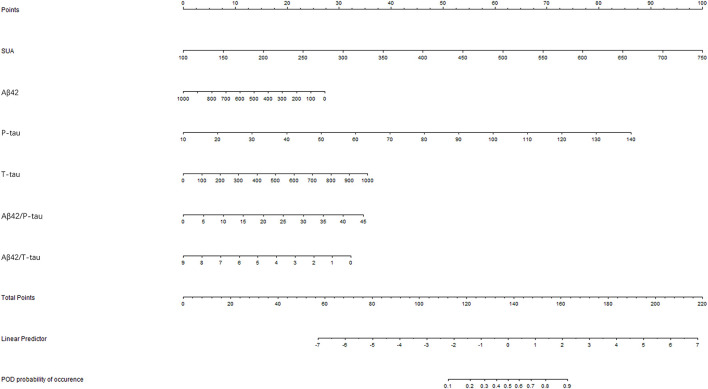
Visualization for predictive factors. Nomogram showed the predictive ability of serum uric acid (SUA) and the Alzheimer-related biomarkers.

Receiver-operating characteristics (ROC) curve showed that the model combining SUA and Alzheimer-related biomarkers (AUC = 0.880; *p* < 0.001) exhibited a relative better discriminatory ability in POD prediction compared to only SUA (AUC = 0.739; *p* < 0.001) (Figure 6A), and the calibration indicated good prediction of it (Figure 6B). The efficacy of each predictor is shown in the nomogram (Figure 7).

## Discussion

In this study, we investigated the relationship between SUA and POD. The results showed that high SUA may increase the risk of POD. This is consistent with the Rotterdam study which suggests that hyperuricemia is associated with white matter atrophy and cognitive decline (Verhaaren et al., 2013; Cervellati et al., 2014; Cicero et al., 2015). Besides, we found that CSF P-Tau was identified as a key mediator of the effect of SUA on POD, and the model combining SUA and Alzheimer-related biomarkers had better prediction efficiency for POD.

As one of the critical pathogenesis, neuroinflammation is thought to be associated with POD (van Gool et al., 2010). Surgery can trigger the release of inflammatory cytokines that penetrate into the brain through the blood–brain barrier (BBB) and induce neuroinflammation (Mattsson et al., 2011; Rochfort and Cummins, 2015). Previous experiments showed that tau protein and Aβ protein had a certain role in POD (Buée et al., 2000; Xie et al., 2014; Chan et al., 2021). Tau protein with abnormal hyperphosphorylation loses its biological function of promoting microtubule assembly and maintaining microtubule stability. Instead, P-tau competes with tubulin to bind normal tau protein and other macromolecule microtubule-related proteins, thus sequestering these proteins from the microtubule and resulting in microtubule depolymerization and the destruction of the normal microtubule system. The deposition of Aβ is not only related to the degeneration of neurons but also capable of activating a series of pathological events, including the activation of astrocytes and microglia, the dysfunction of BBB, and the change in microcirculation. All of these factors affect cognitive function. Based on this study, we found that POD patients had higher preoperative SUA and P-tau levels, while lower preoperative Aβ42 levels.

Although the specific association between uric acid and POD is not completely understood at present, there is some evidence to explain the underlying mechanism. Hyperuricemia has been reported to be associated with white matter atrophy and cerebral ischemia (Schretlen et al., 2007; Verhaaren et al., 2013), and an increased risk of cardiovascular disease (Murray and Burkard, 2016; Wu et al., 2016). In addition, in the process of xanthine oxidoreductase enzyme catalyzing hypoxanthine to produce uric acid (Wang et al., 2014), reactive oxygen species (ROS) are generated, breaking the balance between pro-oxidation and anti-oxidation in the body (Corry et al., 2008). Excessive ROS are well-known to perturb different signaling pathways in a variety of cells (endothelial cells, fat cells, immune cells, etc.), thus leading to various related diseases or diseases. These pathophysiological changes might be explained the potential roles of uric acid in POD pathology. Therefore, further studies are critically needed to clarify the molecular mechanisms.

Besides, a prospective population-based cohort of 4,618 participants aged 55 and older showed that high uric acid was associated with better cognitive function in later life (Euser et al., 2009), and another HABCS study found that high SUA was negatively associated with confidence interval (CI) risk in older adult groups (Chen et al., 2021). This disagreement in previous research may be explained by the following reason. First, the confounding factors adjusted for by each experiment were different. This is especially the case for prospective studies where higher SUA levels were associated with a reduced risk of CI only after adjusting for several cardiovascular risk factors. Second, the basic state of the population is different. The HABCS study included people over 80 years of age, while the Rotterdam study did not exclude patients with cognitive decline or preclinical conditions. Third, differences in race and sample size may also lead to differences in results.

Nonetheless, this study has some limitations. First of all, the follow-up time of the subjects was relatively short, hence the long-term development of the course of the disease remained uncharacterized. Second, the sample size of PNDRFAP and PNDABLE is small, which could be increased for follow-up studies. The inclusion of imaging indicators can help us to better conduct observational studies. In addition to the CSF Alzheimer-related biomarkers, we can also examine the content and changes in plasma biomarkers and use animal models to further explore the mechanism of POD induced by high SUA.

## Conclusion

The present study showed that high SUA may be an independent risk factor for POD. CSF P-tau is a key mediator for the influences of SUA on POD, suggesting that high SUA caused POD mainly by affecting CSF P-tau. The combination of SUA and the CSF Alzheimer-related biomarkers can accurately predict the occurrence of POD in patients. It provides a theoretical basis for clinical prevention and treatment of POD, thus having significant clinical guiding value.

## Data Availability Statement

The raw data supporting the conclusions of this article will be made available by the authors, without undue reservation.

## Ethics Statement

The studies involving human participants were reviewed and approved by the Ethics Committee of Qingdao Municipal Hospital. The patients/participants provided their written informed consent to participate in this study.

## Author Contributions

YB conceived the current study. FW, XT, and JW performed the experiments. SL and XW analyzed the data. RD, XL, and BW performed the experiments and wrote and revised the manuscript. All authors have contributed to the manuscript by revising and editing critically for important intellectual content and given final approval of the version and agreed to be accountable for all aspects of the work presented here. All authors read and approved the final manuscript.

## Funding

This work was supported by the National Natural Science Foundation Youth Project (Grant No. 91849126) and the B. Braun Anesthesia Research Fund (Grant No. BBDF-2019-010).

## Conflict of Interest

The authors declare that the research was conducted in the absence of any commercial or financial relationships that could be construed as a potential conflict of interest.

## Publisher's Note

All claims expressed in this article are solely those of the authors and do not necessarily represent those of their affiliated organizations, or those of the publisher, the editors and the reviewers. Any product that may be evaluated in this article, or claim that may be made by its manufacturer, is not guaranteed or endorsed by the publisher.
